# Anti-GPC3-CAR T Cells Suppress the Growth of Tumor Cells in Patient-Derived Xenografts of Hepatocellular Carcinoma

**DOI:** 10.3389/fimmu.2016.00690

**Published:** 2017-01-11

**Authors:** Zhiwu Jiang, Xiaofeng Jiang, Suimin Chen, Yunxin Lai, Xinru Wei, Baiheng Li, Simiao Lin, Suna Wang, Qiting Wu, Qiubin Liang, Qifa Liu, Muyun Peng, Fenglei Yu, Jianyu Weng, Xin Du, Duanqing Pei, Pentao Liu, Yao Yao, Ping Xue, Peng Li

**Affiliations:** ^1^State Key Laboratory of Respiratory Disease, Guangzhou Institutes of Biomedicine and Health, Chinese Academy of Sciences, Guangzhou, China; ^2^Key Laboratory of Regenerative Biology, South China Institute for Stem Cell Biology and Regenerative Medicine, Guangzhou Institutes of Biomedicine and Health, Chinese Academy of Sciences, Guangzhou, China; ^3^Guangdong Provincial Key Laboratory of Stem Cell and Regenerative Medicine, South China Institute for Stem Cell Biology and Regenerative Medicine, Guangzhou Institutes of Biomedicine and Health, Chinese Academy of Sciences, Guangzhou, China; ^4^Department of Surgery, The Second Affiliated Hospital of Guangzhou Medical University, Guangzhou, China; ^5^Luogang Chinese Medicine Hospital, Guangzhou, China; ^6^Guangdong Zhaotai InVivo Biomedicine Co. Ltd, Guangzhou, China; ^7^Department of Hematology, Nanfang Hospital, Southern Medical University, Guangzhou, China; ^8^Department of Thoracic Oncology, The Second Xiangya Hospital of Central South University, Changcha, China; ^9^Department of Hematology, Guangdong Provincial People’s Hospital, Guangzhou, China; ^10^Wellcome Trust Sanger Institute, Cambridge, UK

**Keywords:** cell therapy, T cells, CAR, hepatocellular carcinoma, PDX

## Abstract

**Background:**

The lack of a general clinic-relevant model for human cancer is a major impediment to the acceleration of novel therapeutic approaches for clinical use. We propose to establish and characterize primary human hepatocellular carcinoma (HCC) xenografts that can be used to evaluate the cytotoxicity of adoptive chimeric antigen receptor (CAR) T cells and accelerate the clinical translation of CAR T cells used in HCC.

**Methods:**

Primary HCCs were used to establish the xenografts. The morphology, immunological markers, and gene expression characteristics of xenografts were detected and compared to those of the corresponding primary tumors. CAR T cells were adoptively transplanted into patient-derived xenograft (PDX) models of HCC. The cytotoxicity of CAR T cells *in vivo* was evaluated.

**Results:**

PDX1, PDX2, and PDX3 were established using primary tumors from three individual HCC patients. All three PDXs maintained original tumor characteristics in their morphology, immunological markers, and gene expression. Tumors in PDX1 grew relatively slower than that in PDX2 and PDX3. Glypican 3 (GPC3)-CAR T cells efficiently suppressed tumor growth in PDX3 and impressively eradicated tumor cells from PDX1 and PDX2, in which GPC3 proteins were highly expressed.

**Conclusion:**

GPC3-CAR T cells were capable of effectively eliminating tumors in PDX model of HCC. Therefore, GPC3-CAR T cell therapy is a promising candidate for HCC treatment.

## Introduction

Hepatocellular carcinoma (HCC) accounts for 90% of primary liver cancers and is one of the deadliest cancers in Asia (1–3). Current curative approaches for liver cancer mainly involve partial liver resection, liver transplantation, chemotherapy, and transarterial chemoembolization (4, 5). Despite enormous advances in the diagnosis and treatment of liver cancer in the recent decades, the 5-year survival rate has remained at about 10% (6, 7). Thus, more novel potential strategies, such as immunotherapy with genetic engineering of T cells to express chimeric antigen receptor (CAR), are now being tested in clinical trials (http://www.clinicaltrials.gov). For accelerating the steps of clinical trials, careful preclinical evaluations in models that closely mirror the clinical situation are urgently required.

Patient-derived xenografts (PDXs) refer to a procedure in which cancerous tissue from a patient’s primary tumor is implanted directly into an immunodeficient mouse (8). This technique offers several advantages over standard cell line xenograft models. Unlike cancer cell lines, primary tumor cells are directly derived from human tissues and are not subjected to frequent high-serum environments and passages. Thus, PDX models are more biologically stable when passaged in mice in terms of mutational status, gene expression patterns, drug responsiveness, and tumor heterogeneity (9). Despite these benefits, only two studies report the use of PDX models of HCCs in drug testing (10, 11). No study has yet examined the use of CAR T cells in PDX models of HCC. Thus, it is necessary to carry out preclinical evaluation of novel CAR T cells against HCCs in PDX models.

It has been shown that glypican-3 (GPC3), a 580-AA heparan sulfate proteoglycan, expresses in 75% of HCC samples but not in healthy liver or other normal tissue (12). GPC3 is, therefore, a suitable target for CAR T cell therapy. Two previous studies showed the promising activity of GPC3-CAR T cells against HCC cell lines *in vivo* (13, 14). However, the capacity of GPC3-CAR T cells to eliminate HCC has not been evaluated in PDX models yet. In this study, we established and characterized primary human HCC xenografts to assess the cytotoxicity of adoptive GPC3-CAR T cells.

## Materials and Methods

### Establishment of HCC Xenografts

Written informed consent was obtained from 12 patients, and the study received ethics approval from the Research Ethics Board of GIBH and the Second Affiliated Hospital of Guangzhou Medical University. All experimental protocols were performed in accordance with guidelines set by the China Council on Animal Care and the Ethics Committee of Animal Experiments at GIBH. The mice were provided with sterilized food and water *ad libitum* and housed in negative pressure isolators with 12-hour light/dark cycles. The isolation was performed following a previously described method with some modifications. The diagnosis of HCC was confirmed by histologic analysis in all cases. HCC tissues were transplanted into NOD/SCID/IL2rg^−/−^ (NSI) mice that were sourced from Li’s lab (15–17). Primary HCC tumors were placed in RPMI 1640 in an ice bath. Thin slices of tumor were diced into ~25 mm^3^ pieces. The tissue was transplanted subcutaneously in the right flank of 8-week-old male NSI mice. Growth of the established tumor xenografts was monitored at least twice weekly through measurement of the length (a) and width (b) of the tumor. The tumor volume was calculated as (*a* × *b*^2^)/2. For serial transplantation, tumor-bearing animals were anesthetized with diethyl ether and sacrificed *via* cervical dislocation. Tumors were minced under sterile conditions and transplanted in successive NSI mice as described earlier.

For the Huh-7 and HepG2 xenograft model, mice were inoculated subcutaneously with 2 × 10^6^ Huh-7 cells on the right flank. When the tumor volume was approximately 50–100 mm^3^, the xenografts were randomly allocated into two groups, and the mice were given intravenous injection of human GPC3-CAR T or Control-CAR T cells in 200-µL phosphate-buffered saline solution as indicated. The tumor volume was calculated as (*a* × *b*^2^)/2.

### Genes and Lentiviral Vectors

To generate CARs-targeting GPC3, the genes of anti-GPC3 scFv, based on GC33 antibodies (18) and anti-CD19 scFv as Control ScFv, were first synthesized and subcloned in frame into lentiviral vectors containing expression cassettes encoding an IgM signal peptide and CD3ζ, CD28ζ, and 4-1BBζ signaling domains under the control of an EF-1α promoter. The sequence of each cloned CAR was verified *via* sequencing.

### Cell Lines and Reagents

A total of 293 T cells were used for lentivirus production and were cultured with DMEM (Gibco, Life Technologies), supplemented with 10% fetal bovine serum (FBS), 2 mM l-glutamine, 50 µM β-mercaptoethanol, 100 IU/mL of penicillin, and 100 IU/mL of streptomycin. HepG2 (HB-8065, purchased from ATCC), Huh-7 (gifted from Dr. Xiaoping Chen, GIBH), and A549 (CCL-185, purchased from ATCC) were transduced with a lentiviral vector co-expressing GFP and luciferase. HepG2-GL (HCC line, stably transfected with GFP and luciferase), Huh7-GL (HCC line, stably transfected with GFP and luciferase), and A549-GL (lung adenocarcinoma line, stably transfected with GFP and luciferase) cells were cultured with DMEM (Gibco, Life Technologies) supplemented with 10% FBS, 2 mM l-glutamine, 50 µM β-mercaptoethanol, 100 IU/mL of penicillin, and 100 IU/mL of streptomycin. Human recombinant interleukin (IL)-2 was obtained from Peprotech. Polyethylenimine, an efficient transfection agent, was purchased from Life Technologies. Anti-GPC3 and anti-AFP were purchased from Santa Cruz Biotechnology, anti-CD3 (BV421) from Biolegend, and the remainder from eBioscience: CD45RO (Clone UCHL1), CD38 (clone HIT2), CD45 (clone HI30), CD19 (clone HIB19), CD5 (clone UCHT2), CD137 (clone 4B4-1), CD62L (clone DREG-56), CCR7 (clone 3D12), CD3 (clone OKT3), CD86 (clone IT2.2), PD-1 (clone eBioJ105), CD44 (clone IM7), TIM3 (clone F38-2E2), CD25 (clone BC96), CD49d (clone 9F10), CD18 (clone 6.7), CD27 (clone O323), CD163 (clone eBioGHI/61), CD326 (clone 1B7), CD66b (clone G10F5), CD3 (clone WM-59), CD206 (clone 19.2), CD80 (clone16-10A1), CD24 (clone eBioSN3), CD42b (clone HIP1), CD36 (clone eBioNL07), CD127 (clone eBioRDR5), LAG3 (clone 3DS223H), CD107a (clone eBioH4A3), CTLA4 (clone 14D3), CD28 (clone CD28.2), CD56 (clone TULY56), CD49f (clone eBioGoH3), HLA-DR (clone L243), CD4 (clone OKT4), and CD8 (clone OKT8).

### Isolation, Transduction, and Expansion of Primary Human T Lymphocytes

Peripheral mononuclear cells (PBMCs) were separated *via* density gradient centrifugation (Lymphoprep, Stem Cell Technologies, Vancouver, BC, Canada). Primary human T cells were isolated from PBMCs *via* negative selection using the pan T Isolation Kit (Miltenyi Biotec, Germany). T cells were cultured in RPMI 1640 supplemented with 10% FCS (Gibco, Life Technologies), 100-U/mL penicillin, and 100 g/mL streptomycin sulfate (R10) and were stimulated with particles coated with anti-CD3/anti-CD28 antibodies (Miltenyi Biotec, Germany) at a cell-to-bead ratio of 1:2. Approximately 72 h after activation, T cells were transfected with supernatant containing lentiviral vectors expressing Control or GPC3-CARs. After transduction for 12 h, T cells were cultured with R10 medium supplemented with IL-2 (300 IU/mL). T cells were fed with fresh media every 2 days and were used within 21 days of expansion in all experiments.

### Cytotoxicity Assays

The target cells HepG2-GL, Huh-7-GL, and A549-GL were incubated with Control-CAR T or GPC3-CAR T cells at the indicated ratios in triplicate wells in U-bottomed, 96-well plates. Target cell viability was monitored 24 h later by adding 100 µL/well substrate d-Luciferin (potassium salt; Cayman Chemical, USA) resolved at 150 µg/mL. The background luminescence was negligible (<1% the signal from the wells with only target cells). The viability percentage (%) was, therefore, equal to the experimental signal/maximal signal, and the killing percentage was equal to 100 − viability percentage.

### Enzyme-Linked Immunosorbent Assay (ELISA)

Enzyme-Linked Immunosorbent Assay kits for IL-2 and interferon-γ were purchased from eBioscience, San Diego, CA, USA, and all ELISAs were conducted in accordance with the manuals provided. Control-CAR T and GPC3-CAR T cells were co-cultured at a 1:1 *E*/*T* ratio for 24 h in duplicate wells, from which the supernatant was collected and measured for the concentrations of IL-2 and IFN-γ.

### Quantitative Real-Time Polymerase Chain Reaction (PCR)

mRNA was extracted from cells with TRIzol reagent (Qiagen, Stockach, Germany) and reverse transcribed into cDNA using the PrimeScript™ RT reagent Kit (Takara, Japan). All reactions were performed with TransStart Tip Green qPCR SuperMix (TransGene, Beijing, China) on a Bio-Rad CFX96 real-time PCR machine (Bio-Rad, Hercules, CA, USA), using the primers shown in Table S1 in Supplementary Material. Delta CT calculations were relative to β-actin and corrected for PCR efficiencies.

### Flow Cytometry

Flow cytometry for the GFP% of transduced T cells and for GPC3 and PD-L1 expression on HCC cells was performed on a C6 cytometer and analyzed using the FlowJo software. The PBMCs, spleens, and bone marrow (BM) from xenograft mice were treated with a red blood cell lysis buffer (Biolegend), and the cells were stained with anti-hCD3, hCD4, and hCD8 analyzed on a Fortessa cytometer (BD Biosciences). All FACS staining was performed on ice for 30 min and washed with PBS containing 2% FBS before cell cytometry. Mouse tissues were weighed and harvested into ice-cold RPMI 1640. The tissues were manually morselized with a scalpel and then mechanically disaggregated through 40- to 100-mm filters.

### Histological Analysis

Organ or tissue samples were fixed in 10% neutral formalin, embedded in paraffin, sectioned at 4-µm thickness, and stained with hematoxylin and eosin or antibodies (GPC3 and AFP). Images were obtained on a microscope (Leica DMI6000B, Leica Microsystems, Wetzlar, Germany).

### Statistics

The data are presented as the mean ± SEM. The results were analyzed *via* an unpaired Student’s *t*-test (two-tailed). Statistical significance was defined by a *P* value of less than 0.05. All statistical analyses were performed using the Prism software version 6.0 (GraphPad).

## Results

### Establishment and Characterization of HCCs from PDXs

**Figure 1:**
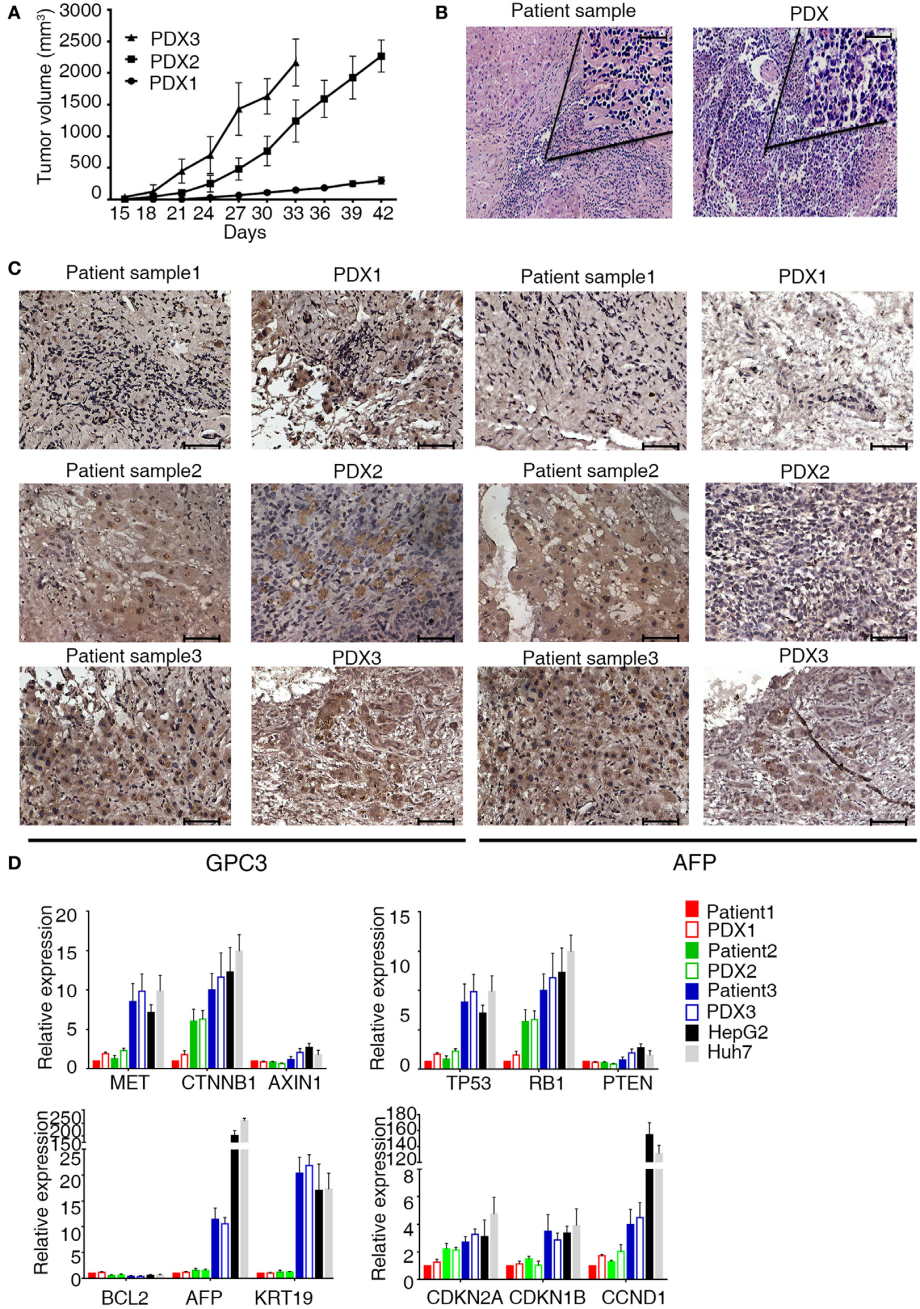
**Characterization of patient-derived xenograft (PDX) model of hepatocellular carcinoma**. **(A)** Patient-derived xenografts (PDXs) were established, and tumor growth was measured at a given time for each PDX xenograft. Tumor volume was calculated as (*a* × *b*^2^)/2, the length (a), and width (b) of tumor. **(B,C)** H&E and IHC staining tissue of primary tumor and PDXs. The xenograft demonstrates morphology, glypican 3, and AFP staining consistent with the original human tumor. Brown color indicates positive staining. IgG was used as a negative control. Scale bar is 50 μm. **(D)** mRNA expression level of tumor-related genes [MET (MET proto-oncogene, receptor tyrosine kinase), CTNNB1 (catenin beta 1), AXIN1 (axin 1), TP53 (tumor protein p53), RB1 (RB transcriptional corepressor 1), PTEN (phosphatase and tensin homolog), BCL2 (BCL2, apoptosis regulator), AFP (alpha fetoprotein), KRT19 (keratin 19), CDKN2A (cyclin-dependent kinase inhibitor 2A), CDKN1B (cyclin-dependent kinase inhibitor 1B), and CCND1 (cyclin D1)] in primary tumor and PDXs. Results represent mean ± SD of three individual experiments.

Of the 12 models, 6 did not grow in the first generation (P1). PDXs of HCC were successfully engrafted from six PDXs in immunodeficient mice (NSI, NOD/SCID-IL2rg^−/−^). Of these, three xenografts were propagated beyond the third generation (P3) (Figure 1A), whereas three tumors were still growing in the first generation (P1). Taken together, a success rate of 25% was reached when the third generation was considered successfully engrafted. An overview of the successfully growing PDX models and their clinical characteristics of the original patients are shown in Table 1.

**Table 1:** **Clinical information of patients**.

HCC	Sex	Age (years)	Site	HBsAg	Metastasis	Histologic grade	PD-L1	AFP (UI/mL)	Xenografts
Patient 1	Male	47	Left lobe	−	No	III	−	4	3rd
Patient 2	Female	47	Right lobe	+	No	IV	−	2.15	3rd
Patient 3	Male	53	Right lobe	+	Intrahepatic	IV	+	384.99	3rd
Patient 4	Male	50	Right lobe	+	No	III	−	3.85	1st
Patient 5	Male	60	Right lobe	+	No	III	+	1.68	1st
Patient 6	Male	67	Left lobe	−	No	III	−	62.54	1st

To validate the established PDX models, we compared their morphology, immunological markers (GPC3 and AFP), and gene expressions with those of the corresponding primary tumor. Histologic evaluation of the xenografts revealed tumor tissue with morphologic characteristics like those of the original primary human tumor (Figure 1B). Immunologic markers of liver tumors such as GPC3 and APF were detected in both primary patient tumors and xenografts (Figure 1C). Quantitative reverse transcription PCR was performed to characterize the mRNA expression level of some tumor-related genes in xenografts and primary tumor (Figure 1D). These genes are associated with carcinogenesis, aggression, and characterization of HCCs (19, 20). Unsupervised hierarchical clustering of selected transcriptional profiles confirmed that all patients and xenograft pairs cluster together (Figure S1 in Supplementary Material). Collectively, our results indicate that PDX of HCCs in the mice recapitulate the original disease and remain stable through three serial transplantations.

### T Cells Engineered to Express GPC3-CARs

**Figure 2:**
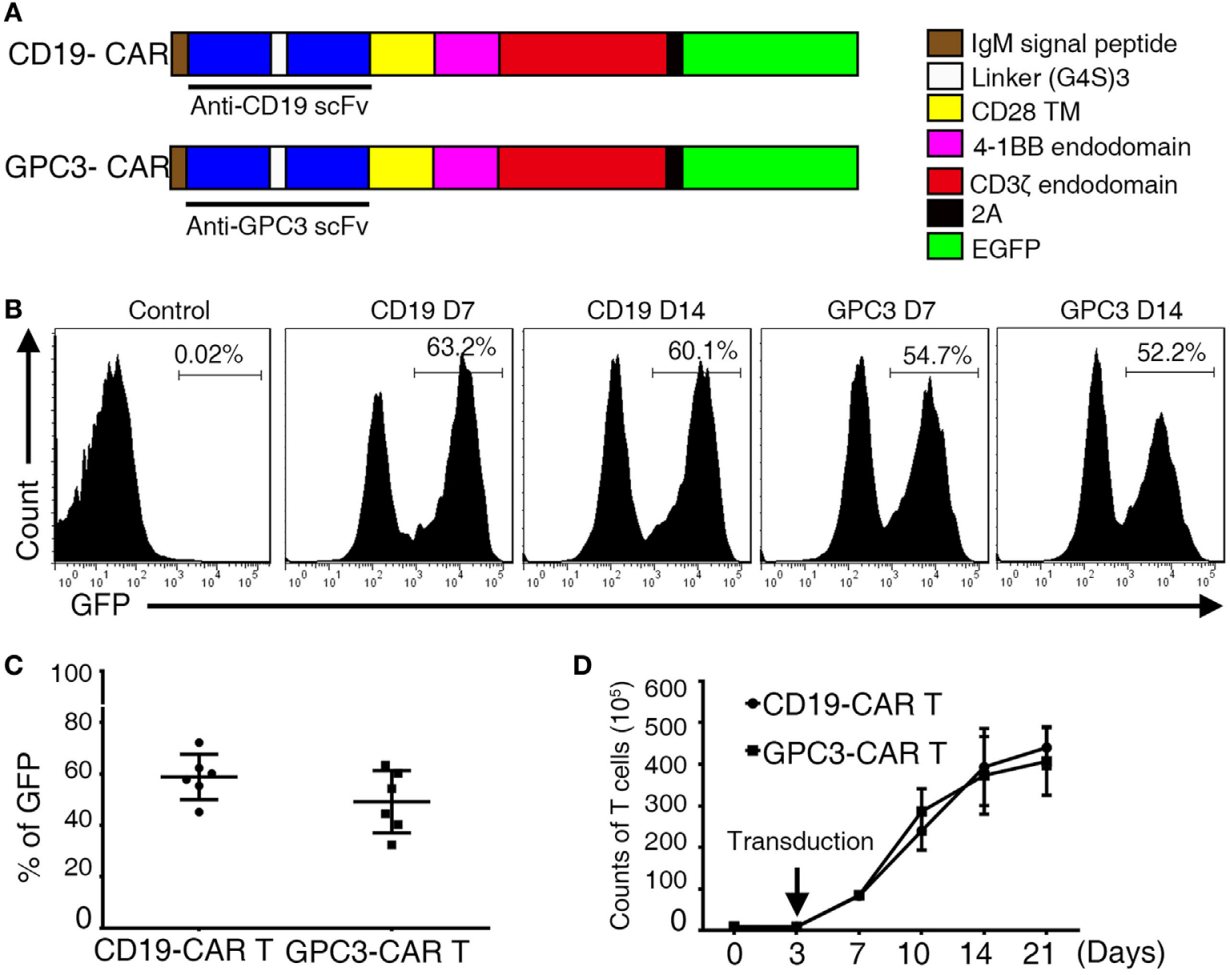
**Construction of chimeric anti-glypican 3 (GPC3) vectors and generation of GPC3-chimeric antigen receptor (CAR) T cells**. **(A)** Schematic representation of a lentiviral vector encoding the signal peptide, anti-GPC3 scFv, CD28 transmembrane domain, 4-1BB costimulatory endodomain, and CD3ζ signaling domain along with eGFP using 2A. **(B)** A representative of GPC3-CARs expression on human T cells transduced with lentivirus was analyzed using flow cytometry, which detected eGFP at days 7 and 14. **(C)** Transduction efficiency. Results represent mean ± SD of six individual experiments. No difference was detected between the percentages of GPC3-CAR T and Control CAR T. **(D)** The expansion of transduced T cells *in vitro* from day 0 to 21. Results represent mean ± SD of three individual experiments.

The sequencing encoding the anti-GPC3 scFv (Figure S2A in Supplementary Material) was cloned in frame into lentivirus vectors containing CAR expression cassettes with CD28, 4-1 BB, and CD3ζ endodomains (Figure 2A). For the generation of T cell populations that expressed the anti-hGPC3-CAR, a two-step optimal expansion protocol was developed. CD3- and CD28-activated T cells after 72 h were transduced with the GPC3-CAR construct to generate GPC3-CAR T cells. The expression of CARs was measured *via* flow cytometry through eGFP expression. CARs were stably expressed from day 7 to day 14 with no significant difference (Figure 2B). The frequency of CAR expression was 58.6% for CD19-CAR and 49.2% for GPC3-CAR (Figure 2C). Flow cytometric analysis using a goat anti-mouse F(ab)2 confirms that the expression of CAR molecules was consistent with eGFP (Figure S2B in Supplementary Material). The generated CAR T cells were >97% CD3-positive T cells, which consisted of CD4- and CD8-positive T cell subsets with the same ratio as the non-transduced T cells (Figures S3A–D in Supplementary Material). In our optimal expansion protocol, T cells begin to expand at day 3 and continued to expand until day 21. Reproducible expansion of 20- to 50-folds of T cell can be achieved at day 14 (Figure 2D). Collectively, these experiments established a robust two-step method to transduce and expand (up to 50-fold) CAR-transduced T cells from the peripheral blood of healthy donors.

### Phenotypic and Functional Characterization of GPC3-CAR T Cells

**Figure 3:**
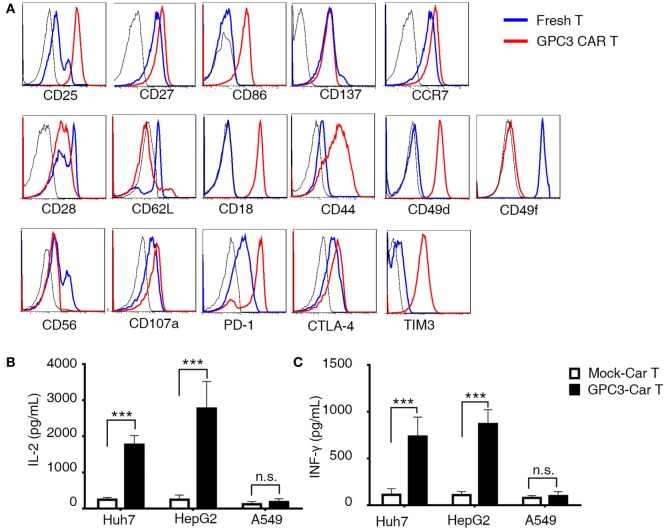
**Phenotypic analysis and cytokines produced in glypican 3 (GPC3)-chimeric antigen receptor (CAR) T cells**. **(A)** Flow cytometry comparison of the common surface phenotype of GPC3-CAR T cells (red line) at day 14 of culture with freshly isolated T cells (blue line). Black line represents the isotype control. Histogram overlays show 16 markers related to lymphocyte activation, differentiation, migration, adhesion, and exhaustion. **(B)** Interferon-γ and **(C)** interleukin-2 were secreted by the indicated modified CAR T cells co-cultured with hepatocellular carcinoma cell lines and A549 for 24 h. Results represent triplicates. ***P < 0.001 with *T*-test.

To better define the phenotyping of CAR-transduced T lymphocytes after infection, we next performed 35 different cell surface markers. CAR T cells were compared at the beginning (day 0) and the middle (day 14) of the T cell culture process. We observed upregulation of the activation markers CD25 and CD27, the migration maker CCR7 (Figure 3A), and the costimulatory receptors CD86 (21) and CD137 (Figure 3A), which are indicators of enhanced proliferative potential of T cells. After *in vitro* culture, T cells acquired an intermediate effector memory phenotype with the progressive downregulation of CD28 and CD62L. Moreover, we observed upregulation or downregulation of multiple molecules involved in cell adhesion. CD18, CD44, and CD49d were upregulated, and CD49f, CD107a, and CD56 were downregulated (Figure 3A). Notably, key inhibitory and exhaustion-associated molecules such as PD-1, CTLA-4, and TIM3 were upregulated (Figure 3A). Importantly, we found strikingly similar CAR T cell phenotypes across all six tested donors, as illustrated in the heat map in Figure S3E in Supplementary Material. A hallmark function of activated T lymphocytes is the production of cytokines. To evaluate this production, we co-cultured CAR T cells with GPC3-positive HCC cell lines as target cells (Figure S4A in Supplementary Material). GPC3-CAR T cells secreted high levels of INF-γ and IL-2 after coincubation with only GPC3-positive targets (Figures 3B,C). These data collectively characterize CAR T cells as a highly reproducible cellular product of activated lymphocytes, endowed with migratory potential and natural cytotoxic machinery.

### Effective Serial Killing of GPC3-Positive Human HCC Cells by GPC3-CAR T Cells

**Figure 4:**
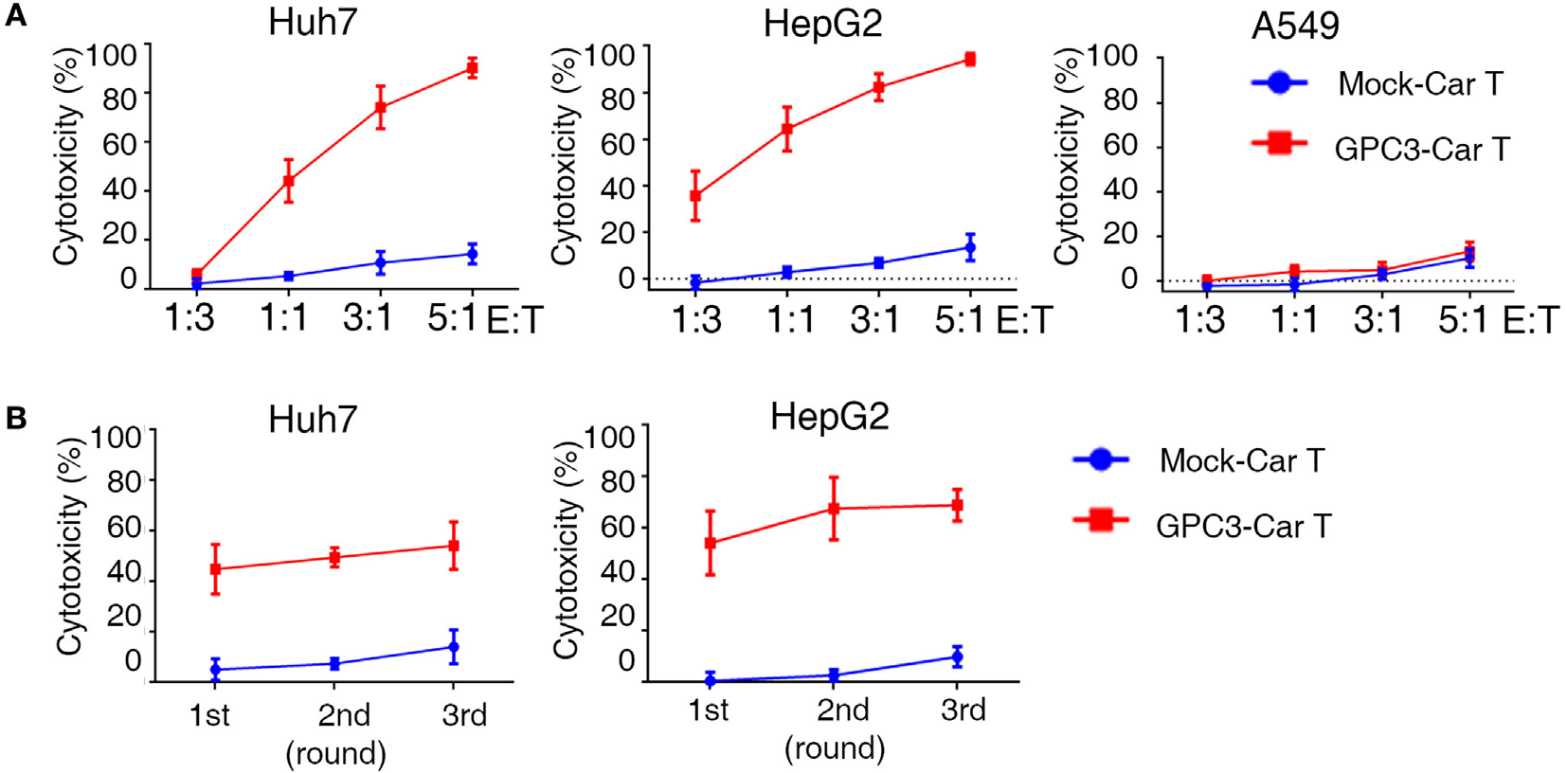
**Cytotoxicity of glypican 3 (GPC3)-chimeric antigen receptor (CAR) T cells against targeted cells *in vitro***. **(A)** Cytotoxicity of GPC3-CAR T cells was assayed by coincubation with Huh-7-GL (GPC3^+^), HepG2-GL (GPC3^+^), and A549-GL (GPC3^−^) cells at E:T ratios ranging from 1:3 to 5:1. **(B)** Serial killing of GPC3-CAR cells against Huh-7-GL and HepG2-GL cells at E: T ratios of 1:1 in indicated durations. Results represent mean ± SEM.

To test whether GPC3-CAR T cells could specifically recognize and kill GPC3-positive targets, cytotoxicity assays were performed by incubating the CAR T cells with GPC3-positive HCC cells (Huh-7and HepG2) and GPC3-negative cells (A549) (Figure S4A in Supplementary Material). GPC3-CAR T cells were highly cytotoxic against the GPC3-positive HCC cells, Huh-7, and HepG2. By contrast, GPC3-CAR T cells did not target GPC3-negtive cells (Figure 4A). These data demonstrate that GPC3-CAR T cells selectively target GPC3-positive tumor cells. It had been demonstrated that 4-1BB endodomains ameliorate exhaustion of CAR T cells (22). To further explore cytotoxic potency of GPC3-CAR T cells incorporating 4-1BB costimulatory domains, we performed a co-culture in which CAR T cells were restimulated with GPC3-positive HCC cells every 24 h for three consecutive days at E:T ratios of 1:1 (23). Killing of Huh-7 and HepG2 hepatoma cells was only observed when GPC3-CAR was reconstituted (Figure 4B). Taken together, these results indicate that GPC3-CART cells displayed significantly specific and efficient cytotoxicity against GPC3-positive target cells.

### Adoptive Transfer of GPC3-CAR T Cells Suppresses HCC Cell Lines Growth *In Vivo*

**Figure 5:**
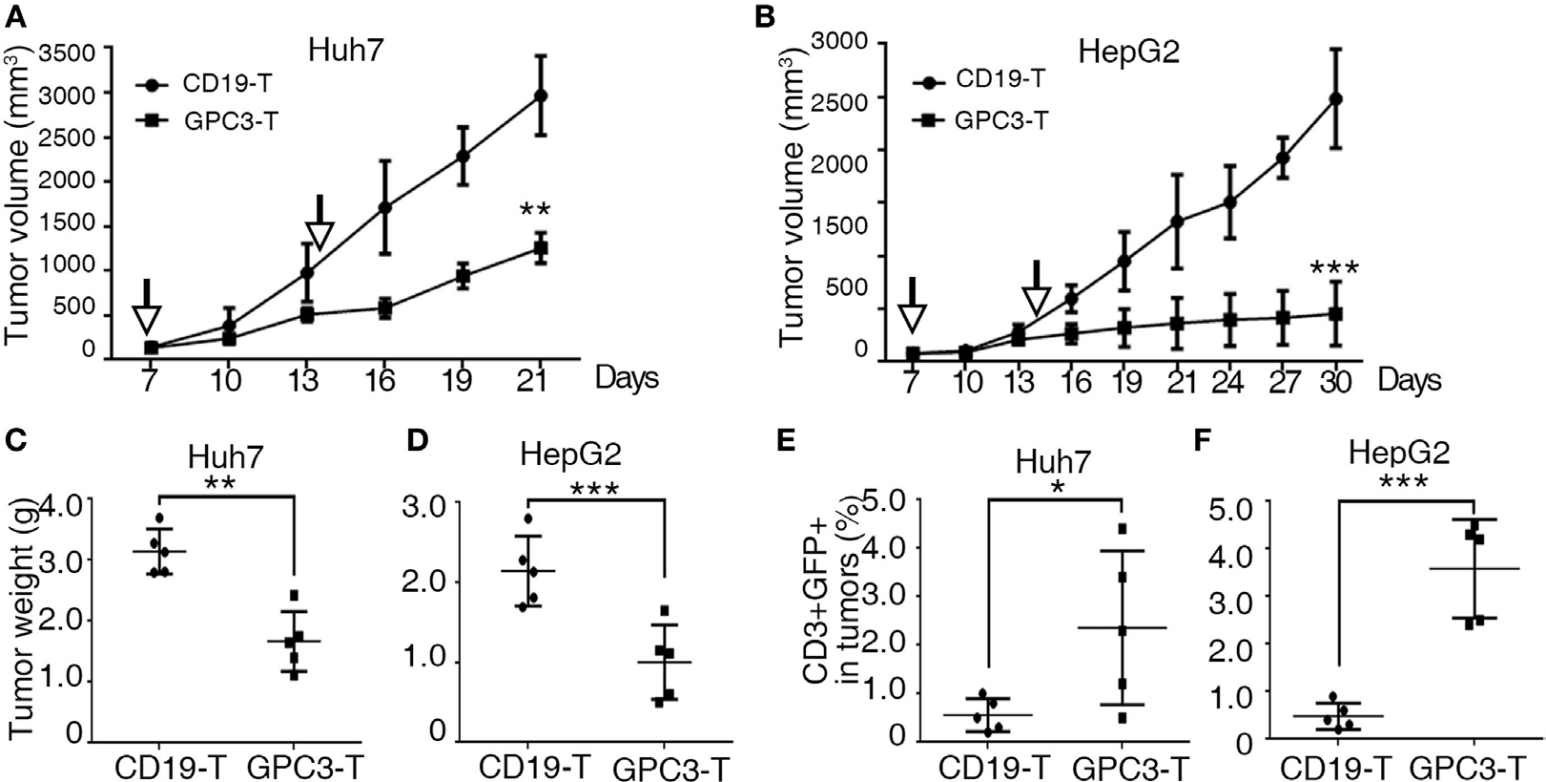
**Cytotoxicity of glypican 3 (GPC3)-chimeric antigen receptor (CAR) T cells against hepatocellular carcinoma cell lines in xenografts**. **(A,B)** Growth curve of Huh-7 and HepG2 xenografts (*n* = 5) treated with the Control- or GPC3-CAR T cells at indicated time point (arrow). At the end of the experiment, the tumors treated with GPC3-CAR T cells were significantly smaller than those in the Control group. **(C,D)** Huh-7 and HepG2 tumor weights from the mice treated with CAR T cells at the end of the experiment, respectively. **(E,F)** GPC3-CAR T cells in tumors were significantly higher than Control-CAR T groups. Results represent mean ± SD. *P < 0.05, **P < 0.01, and ***P < 0.001 with *T*-test.

To explore the killing of GPC3-positive tumors by GPC3-CAR T cells *in vivo*, we used a subcutaneous xenograft model in which transplant tumors were established in immunodeficient mice using HepG2 and Huh-7 cell lines. Tumors were established for 7 days, and a tumor volume of approximately 50–100 mm^3^ was obtained. The mice were then treated *via* adoptive transfer of GPC3-CAR T or Control-CAR T cells. Tumor growth was efficiently suppressed by intravenous injection of 5 × 10^6^ GPC3-CAR T cells (*n* = 5), as compared to a control group that received Control transduced T cells (*n* = 5) (Figures 5A–D). We also detected human T cells in the PBMC and tumor tissues of mice with subcutaneous Huh-7 or HepG2 xenografts after T cell infusion (Figures 5E,F). The results show that GPC3-CAR T cells can efficiently suppress the growth of HCC cell lines in mice.

### Patient-Derived HCC Xenograft Is Controlled by GPC3-CAR T Cells

**Figure 6:**
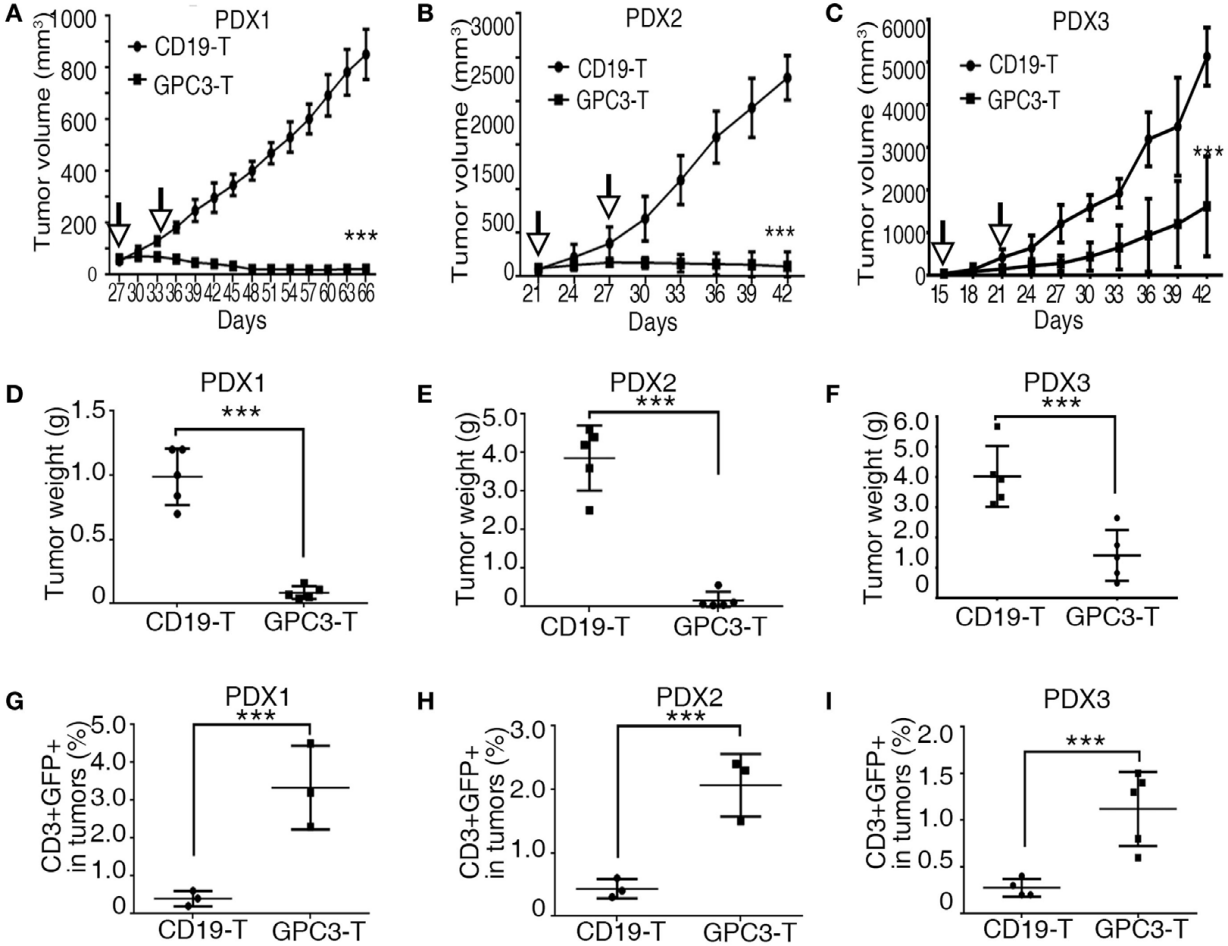
**Glypican 3 (GPC3)-chimeric antigen receptor (CAR) T cells efficiently abolish growth of patient-derived xenografts (PDXs) of hepatocellular carcinoma**. **(A–C)** Growth curve of PDX1, PDX2, and PDX3 (*n* = 5) treated with the Control- or GPC3-CAR T cells at indicated time point (arrow). At the end of the experiment, the tumors treated with GPC3-CAR T cells were significantly smaller than those in the Control group. **(D–F)** PDX1, PDX2, and PDX3 tumor weights from the mice treated with CAR T cells at the end of the experiment. **(G–I)** GPC3-CAR T cells in tumors were significantly higher than Control-CAR T groups. Results represent mean ± SD. ***P < 0.001 with *T*-test.

Patient-derived xenograft models preserve the heterogeneous pathological and genetic characteristics of the original patient tumors and may provide a precision preclinical model for immunotherapy evaluation. Our results show that GPC3 protein was highly expressed in xenografts of HCCs, so we tested the effect of GPC3-CAR T cells in these PDX models. In all three individual PDX models, 2.5 × 10^6^ CAR T cells were given by intravenous injection twice after the tumor volume reached 50–100 mm^3^. The efficient antitumor effect was observed in the xenografts treated with GPC3-CAR T cells compared to the Control-CAR T cells (Figures 6A–F). We observed that GPC3–CAR T cells have better cytotoxicity in PDX1 and PDX2 than PDX3. We propose that the heterogenous nature of tumors can affect GPC3-CAR T cells cytotoxicity to tumors *in vivo*. Studies have shown that the highly expressed MET, CTNNB1, and CCND1 are associated with aggression of HCCs (24). MET, CTNNB1, and CCND1 are highly expressed in PDX3 than in PDX1 and PDX2 (Figure 1D). This result implies that PDX3 tumor cells are more aggressive. Programed cell death 1 (PD-1), an immunoinhibitory receptor belonging to the CD28 family, has been shown as a frequently used physiologic immunosuppressive mechanism by tumors to invade host immunity (25). Our results show that PD-L1 was highly expressed in PDX3 but not in PDX1 and PDX2 (Table 1). These results suggest that tumor aggression and immunosuppressive molecules should be considered in CAR T cell therapy. T cell analysis also shows that GFP-positive T cells are higher in GPC3-CAR T groups than in Control-CAR T groups (Figures 6G–I). Taken together, our results demonstrate that GPC3-CAR T cells were able to efficiently suppress the growth of primary GPC3-positive HCC *in vivo*.

## Discussion

In this study, we report the establishment of three PDX models from primary HCC in NSI mice. The xenografts were successfully serially transplanted while preserving the characteristics of the original patient tumors. We observed that tumors in PDX2 and PDX3 xenografts grow faster than that in PDX1. Our data suggest that their growth behavior is positively correlated with the expression levels of MET, CTNNB1, and CCND1. Previous studies show that MET and CTNNB1 act as oncogenes in HCCs and that CCND1 is the hallmark of cell cycle progression (19, 26, 27).

Previous studies have shown that GPC3-CAR T cells efficiently eradicate liver cancer cells lines that possess a high level of GPC3 expression *in vivo* (13, 14). Here, we observed that GPC3-CAR T cells were less effective to kill HCC cell lines in xenografts, compared to a previous report (13). A possible reason for this difference is that we used NOD/SCID/IL2^−/−^ mice for xenotransplantation, whereas the previous report used NOD/SCID mice, in which natural killer (NK) cells are active against tumor cells. By comparing the subcutaneous growth of Huh-7, we observed that Huh-7 cells grew faster in NOD/SCID/IL2^−/−^ mice than in NOD/SCID mice. In addition, tumor-experienced T cells (28) can promote NK cell activity against tumors cells. It is likely that GPC3-CAR T cells may kill tumor cells in synergy with mouse NK cells.

Patient-derived xenograft models have been commonly used to test drug efficacies and identify biomarkers in a number of cancers, including liver, ovarian, pancreatic, breast, and prostate cancers (9). Previous studies have shown that tumors in PDX models are biologically stable and accurately reflect the histopathology, gene expression, genetic mutations, and therapeutic response of the patient tumor (9). Several recent preclinical studies and clinical trials have demonstrated the efficient activity of CD19-CAR T cells against acute B lymphoblastic leukemia (29). However, CAR T cells that target solid tumors have so far demonstrated limited efficacy. To date, the most positive trials reported have used GD2 CARs to target neuroblastoma (3 of 11 patients with complete remissions), HER2 CARs for sarcoma (4 of 17 patients showing stable disease), and HER1 CARs for lung cancer (2 of 11 patients with partial responses) (30). We report that GPC3-CAR T cells impressively eradicated tumors from PDX1 and PDX2, which were less aggressive and were PD-L1 negative. In contrast, GPC3-CAR T cells were less cytotoxicity to tumors in PDX3 that were more aggressive and highly expressed PD-L1, suggesting that we need to combine CAR T cell therapy and immune checkpoint inhibitors to achieve higher efficacy of eliminating PD-L1-positive HCC. Similarly, two recent reports showed that combining CAR therapy and PD-1 blockade was efficacious in breast cancer and mesothelioma models (31, 32). Downregulated expression of GPC3 in HCC cells may affect GPC3-CAR T-specific cytotoxicity to tumor cells. While our data show that the percentage of GPC3-positive cells was not changed in control and GPC3-CAR T treatment *in vivo* (Figures S4B,C in Supplementary Material).

In summary, we established and characterized three GPC3-positive PDXs of HCC. We also show that GPC3-CAR T cells suppressed tumor growth but with different efficacies in the PDX models of the three individual patients. Therefore, PDX models can potentially be used to evaluate the efficacy of GPC3-CAR T cell therapy for treating HCC in individual patients.

## Author Contributions

ZJ and XJ contributed to the conception and design, collection and/or assembly of data, data analysis and interpretation, and manuscript writing. SC, XW, YL, SL, and QLiang contributed to the provision of study material or patients and collection and/or assembly of data. BL, SW, and QW provided administrative support. YY and DP contributed to the conception and design and provided financial support. YY, QLiu, and PLiu contributed to the conception and design. PX and PLi contributed to the conception and design, data analysis, and interpretation, manuscript writing, and final approval of manuscript and provided financial support. All authors read and approved the final manuscript.

## Conflict of Interest Statement

The authors declare that the research was conducted in the absence of any commercial or financial relationships that could be construed as a potential conflict of interest.
