# Supplementary material for: Anti-GPC3-CAR T Cells Suppress the Growth of Tumor Cells in Patient-Derived Xenografts of Hepatocellular Carcinoma
Source: Front Immunol. 2017 Jan 11;7:690. doi: 10.3389/fimmu.2016.00690 (PMC5225101; doi:10.3389/fimmu.2016.00690)
Supplement: Supplementary file 1 [file Table_1.DOCX]

## Supplementary table 1 Primers used for qRT-PCR.

| Gene | Primer sequence | Fragment Size  (bp) | RefSeq ID |
| --- | --- | --- | --- |
| MET | 5-TGTTCGATATTCATCACGGC-3  5-GCATTTTTACGGACCCAATC-3 | 121 | NM_000245 |
| CTNNB1 | 5- ATTGTCCACGCTGGATTTTC-3  5-AGGTCTGAGGAGCAGCTTCA-3 | 142 | NM_001904 |
| AXIN1 | 5-CCGGCATTGACATAATAGGG-3  5-CCGAGGGTCTCCTCCAGTA-3 | 136 | NM_003502 |
| TP53 | 5-GCTCGACGCTAGGATCTGAC-3  5-GCTTTCCACGACGGTGAC-3 | 97 | NM_00546 |
| RB1 | 5- CCTTCTCGGTCCTTTGATTG-3  5-CAGAAGGCAACTTGACAAGAGA-3 | 130 | [NM_000321](http://www.ncbi.nlm.nih.gov/entrez/query.fcgi?cmd=Search&db=Nucleotide&term=NM_003153) |
| PTEN | 5-CGGTGTCATAATGTCTTTCAGC-3  5-TGAAGGCGTATACAGGAACAAT-3 | 110 | [NM_000314](http://www.ncbi.nlm.nih.gov/entrez/query.fcgi?cmd=Search&db=Nucleotide&term=NM_018441) |
| BCL2 | 5-GAGAAATCAAACAGAGGCCG-3  5-CTGAGTACCTGAACCGGCA-3 | 106 | [NM_000633](http://www.ncbi.nlm.nih.gov/entrez/query.fcgi?cmd=Search&db=Nucleotide&term=NM_004827) |
| AFP | 5-GTGGTCAGTTTGCAGCATTC-3  5-AGAGGAGATGTGCTGGATTG-3 | 110 | [NM_001134](http://www.ncbi.nlm.nih.gov/entrez/query.fcgi?cmd=Search&db=Nucleotide&term=NM_133436) |
| KRT19 | 5-GTCGATCTGCAGGACAATCC-3  5-CCGCGACTACAGCCACTACT-3 | 97 | NC_0022276 |
| CDKN2A | 5-GTGAGAGTGGCGGGGTC-3  5-GTTACGGTCGGAGGCCG-3 | 109 | NC_000077 |
| CDKN1B | 5-TTCATCAAGCAGTGATGTATCTGA-3  5-AAGAAGCCTGGCCTCAGA-3 | 90 | NC_004064 |
| CCND1 | 5-GGCGGATTGGAAATGAACTT-3  5-TCCTCTCCAAAATGCCAGAG-3 | 109 | NC_053056 |
