# Supplementary material for: Anti-GPC3-CAR T Cells Suppress the Growth of Tumor Cells in Patient-Derived Xenografts of Hepatocellular Carcinoma
Source: Front Immunol. 2017 Jan 11;7:690. doi: 10.3389/fimmu.2016.00690 (PMC5225101; doi:10.3389/fimmu.2016.00690)
Supplement: Supplementary file 2 [file Data_Sheet_1.PDF]

Supplementary figure 1

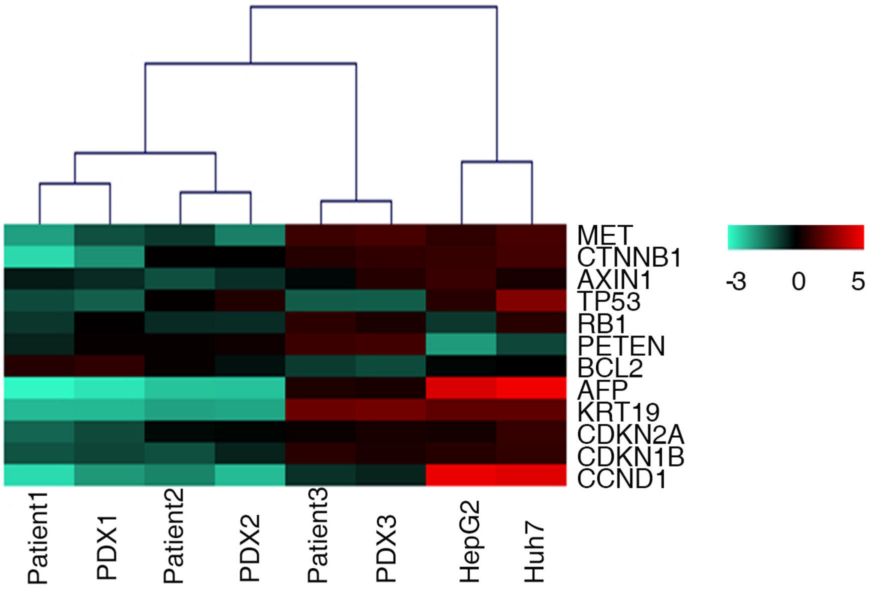

Supplementary figure 2

A

5’-gatgttgtgatgacccaaactccactctccctgcctgtcagtcttggagatcaagcctcca

tctcttgcagatctagtcagagccttgtacacagtaatggaaacacctatttacattggtacct

gcagaagccaggccagtctccaaagctcctgatctacaaagtttccaaccgattttctgggg

tcccagacaggttcagtggcagtggatcagggacagatttcacactcaagatcagcagag

tggaggctgaggatctgggagtttatttctgctctcaaaatacacatgttcctcctacgttcgga

tcggggaccaagctggaaataaaaGGTGGAGGCGGTTCAGGCGGAGG

TGGCAGCGGCGGTGGCGGGTCGcaggttcaactgcagcagtctggggct

gagctggtgaggcctggggcttcagtgaagctgtcctgcaaggcttcgggctacacatttac

tgactatgaaatgcactgggtgaagcagacacctgtgcatggcctaaaatggattggagct

cttgatcctaaaactggtgatactgcctacagtcagaagttcaagggcaaggccacactga

ctgcagacaaatcctccagcacagcctacatggagctccgcagcctgacatctgaggact

ctgccgtctattactgtacaagattctactcctatacttactggggccaagggactctggtcact

gtctctgca-3’

B

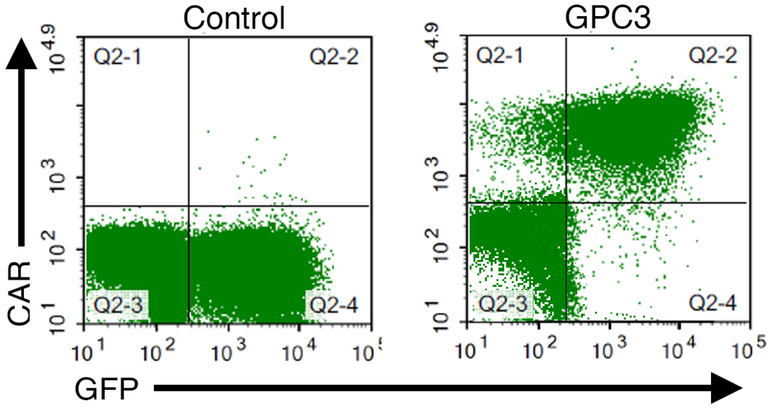

Supplementary figure 3

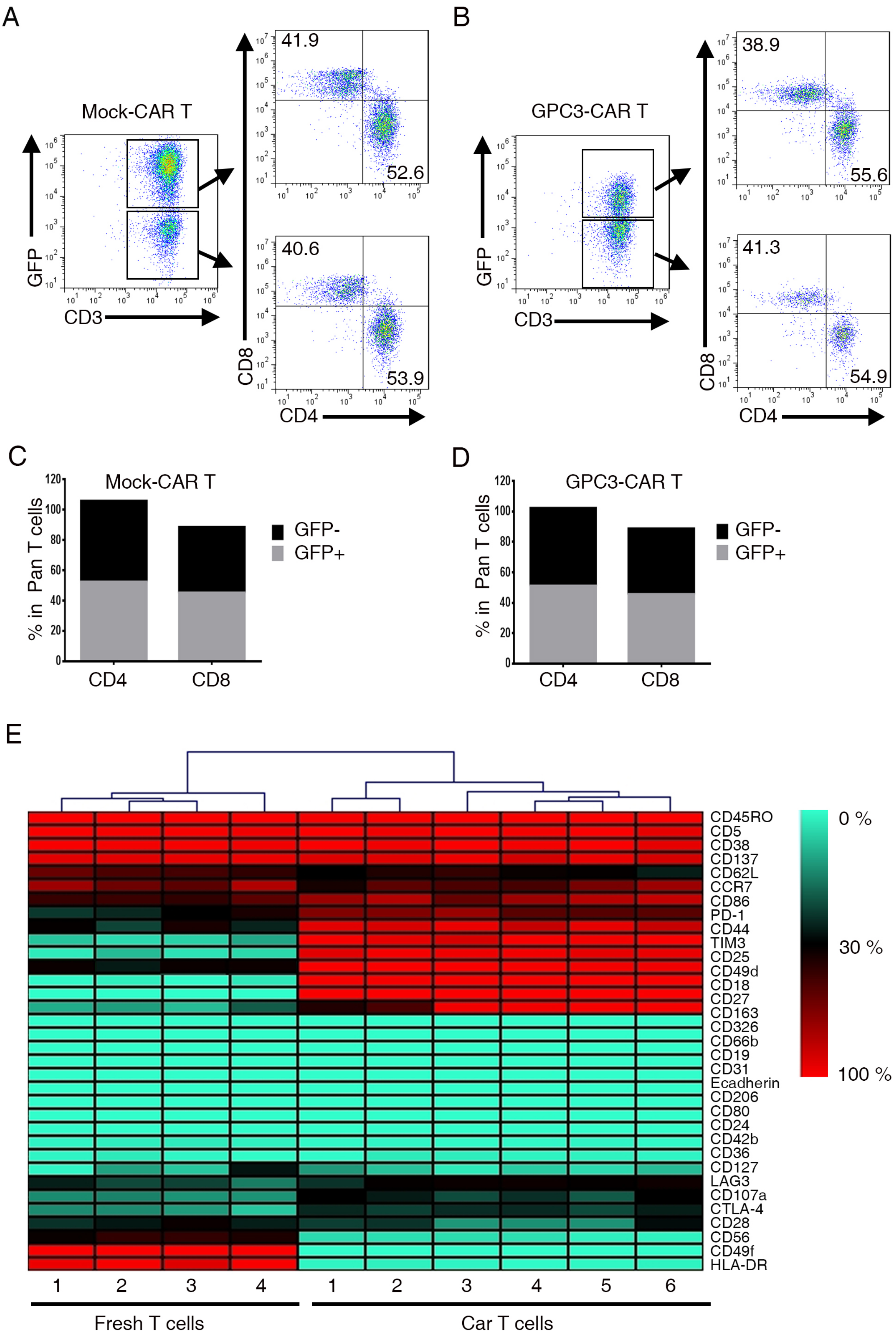

Supplementary figure 4

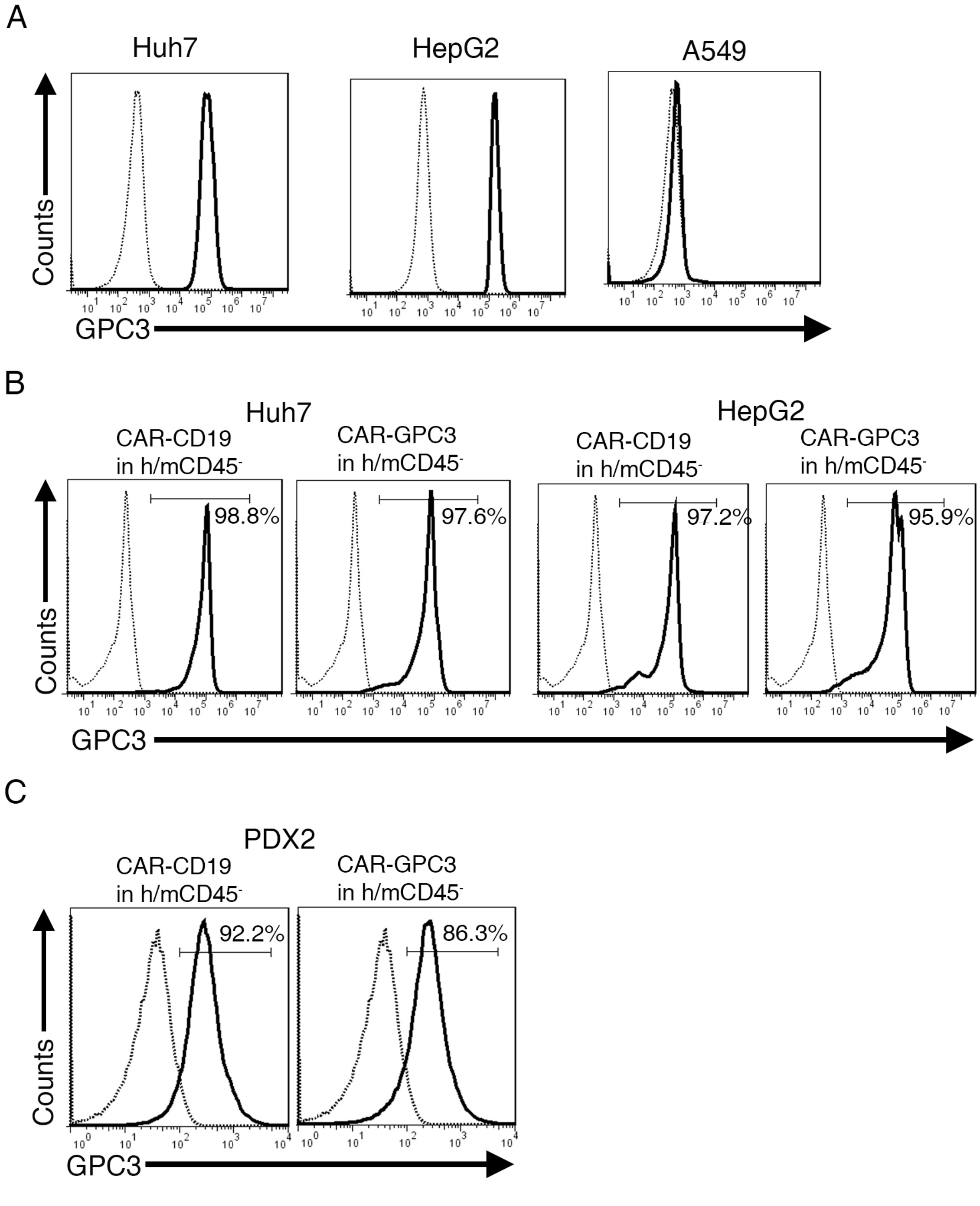

Supplementary figure legend

Supplementary figure 1. Gene expression cluster analysis Unsupervised hierarchical clustering of selected transcriptional profiles confirmed that all patient and xenograft pairs cluster together.

Supplementary figure 2. The encoding sequences of GC33 scFv in the construct of anti-GPC3 CAR (A) Blue encoding sequence: region of light chain of GC33; black encoding sequence: linkers; red encoding sequence: region of heavy chain of GC33. (B) CARs were detected using a goat anti-mouse F(ab)2 in transduced T cells.

Supplementary figure 3. Phenotypic analysis of genetically modified T cells (A, B) Representative FACS analysis of T cells transduced with Mock or GPC3 vectors. (C, D) Quantities of CD4 and CD8 T cells percentage in pan T cell transduced with Mock or GPC3 vectors. (E) Heatmap illustrates percentages of positive cells for each surface marker at day 14 from six individual samples, compared to freshly isolated samples. The color bar is presented on the right.

Supplementary figure 4. GPC3 expression analysis (A) Histogram represents surface GPC3 expression (black line) on Huh7, HepG2, and A549 cell lines detected by flow cytometry and isotype antibody control (dotted line). (B, C) Histogram represents surface GPC3 expression (black line) on Huh7, HepG2, and PDX2 from xenografts treated with CAR T cells. Isotype antibody control (dotted line).
